# Blastic Plasmacytoid Dendritic Cell Neoplasm With Central Nervous System Involvement: A Case Report

**DOI:** 10.7759/cureus.23888

**Published:** 2022-04-06

**Authors:** Diego Molina Castro, Oliver Perilla Suárez, Jorge Cuervo-Sierra, Alexandra Moreno

**Affiliations:** 1 Internal Medicine, Hospital San Vicente Fundación, Medellín, COL; 2 Hematology, Universidad de Antioquia, Medellín, COL; 3 Hematology, Centros Especializados de San Vicente Fundación, Medellín, COL; 4 Hematology, San Vicente Fundación, Rionegro, COL; 5 Hematopathology, Synlab, Medellín, COL

**Keywords:** hematologic neoplasm, new therapies, non-cutaneous bpdcn, venetoclax, blastic plasmacytoid dendritic cell neoplasm

## Abstract

Blastic plasmacytoid dendritic cell neoplasm is a rare hematologic neoplasm characterized by cutaneous, hematologic, and central nervous system (CNS) involvement with poor prognosis. Diagnosis is made by flow cytometry, although there are no specific markers, making its diagnosis challenging. So far, with the available evidence, acute lymphoid leukemia-type schemes and consolidation with allogeneic transplant seem to become the first-line therapy. With its characterization, new therapies directed toward CD123 and the anti-apoptotic protein Bcl-2 have appeared to prolong the survival of these patients. We present a case of a 27-year-old male patient diagnosed with blastic plasmacytoid dendritic cell neoplasm with unusual CNS manifestations and without skin involvement who achieved complete remission with venetoclax and improvement of neurological symptoms, making him a candidate for hematopoietic stem cell transplant.

## Introduction

Blastic plasmacytoid dendritic cell neoplasm (BPDCN) is an extremely rare hematologic neoplasm originating from a subtype of dendritic cells [1]. It occurs more frequently in men over 60 years of age, and its main clinical manifestations are the presence of skin lesions that rapidly evolve and compromise organs such as lymph nodes, bone marrow, viscera, and, to a lesser extent, the central nervous system (CNS) [1]. Diagnosis is based on the identification by flow cytometry (FCM) or immunohistochemistry (IHC) of CD123, CD56, and CD4, whose expression is typical and specific of plasmacytoid blastic dendritic cells [1]. It has an ominous prognosis despite treatment with conventional chemotherapy and even hematopoietic stem cell transplant (HSCT) [2-3]. In recent years, new treatments have appeared to improve survival, mainly based on immunotherapy with targets such as Bcl-2 and CD123 [4].

We present the case of a young patient without skin involvement who debuted with pancytopenia and rapidly presented neurological complications. After a second-line treatment with venetoclax and cytarabine, he achieved complete remission and proceeded to consolidation with HSCT.

## Case presentation

**Figure 1:**
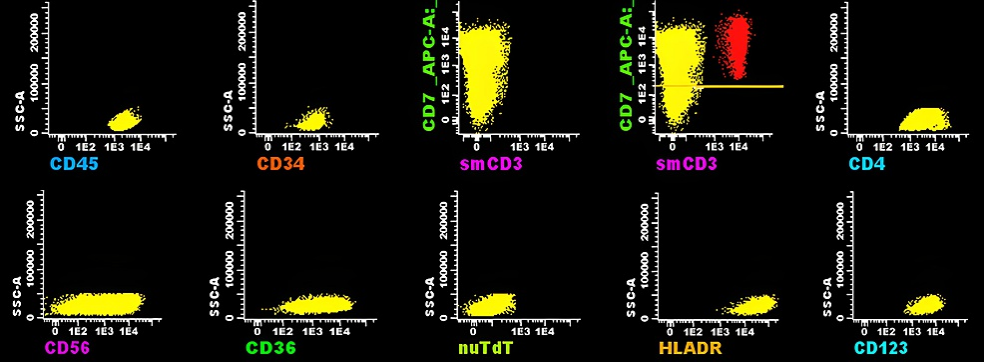
Flow cytometry consistent with blastic plasmacytoid dendritic cell neoplasm Top row (left to right): CD45 and CD34 intermediate, CD3-, CD7 -/++, CD4+. Bottom row (left to right): CD56-/++, CD36+, TdT-, HLA-DR++, CD123+++

A 27-year-old man , workman, with no relevant medical history presented three months of cervical, axillary, and inguinal lymphadenopathies, generalized bone pain, and a weight loss of 8 kg. No fever or night sweats were reported. The physical examination confirmed the presence of generalized, mobile, non-painful lymph nodes with defined borders approximately 1.5 to 2 cm in diameter. No skin lesions or hepatosplenomegaly were found. Laboratory tests revealed anemia, neutropenia, and thrombocytopenia with leukocytosis in peripheral blood smear with cells of blastic characteristics. Lymphoblastic lymphoma versus aggressive lymphoma with leukemic presentation was considered as diagnostic impressions; we performed bone marrow biopsy and excisional biopsy of the cervical lymph node. The FCM of medullary blood showed a pathological population that represented 67.1% with the following phenotype: intermediate CD45, MPO negative (-), CD34 (-), CD117 (-), HLA- DR (+) strong and homogeneous, CD7 (-/+) heterogeneous (positive 80.9%), CD123(+) strong and homogeneous, CD56(+) strong and homogeneous, CD36 (+), CD4 (+), CD38 (-/+) heterogeneous, TdT (-), cCD3 (-), sCD3 (-), CD19 (-), CD79a (-), CD35 (-), CD15 (-), CD64 (-), CD13 (-), CD11b (-), CD16 (-), CD10 (-), CD14 (-), and CD300e (-), marker expression altogether conclusive for BPDCN (Figure 1) and not lymphoblastic lymphoma or aggressive lymphoma with leukemic presentation.

**Figure 2:**
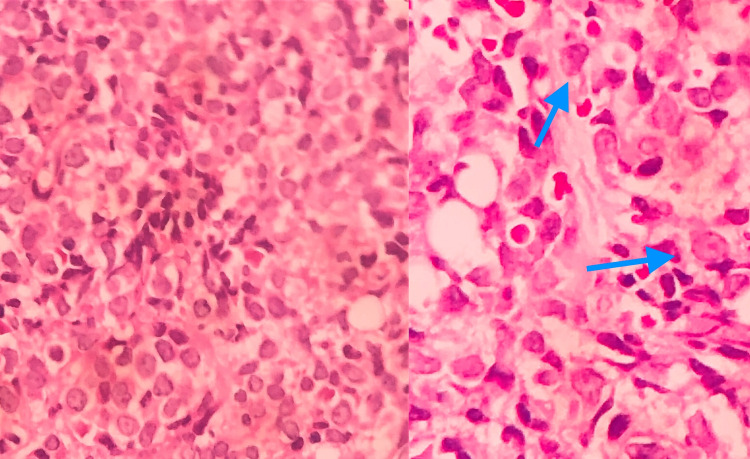
Lymph node cervical biopsy Patchy infiltration of immature-looking monomorphic intermediate-sized cells (left). Both arrows on the right show irregular nucleus, fine chromatin, and evident nucleolus.

The karyotype was 44-46, XY, gain (1) (p36)(cp25) with addition to genetic material on the short arm of chromosome 1 in all metaphases. The final histopathological result of the cervical lymph node showed altered architecture by infiltration of cells compatible with BPDCN. (Figure 2). FCM of cerebrospinal fluid (CSF) also detected the presence of BPDCN.

**Figure 3:**
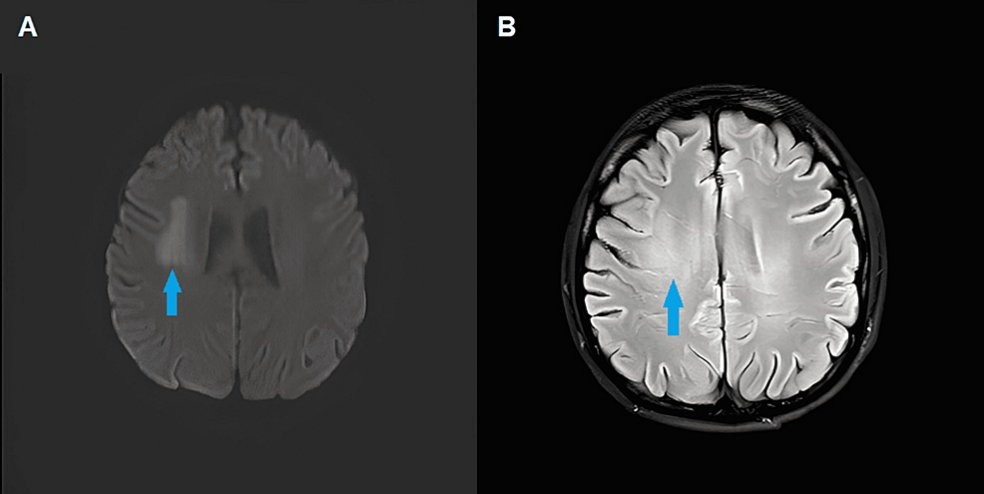
Brain MRI (A) Restricted diffusion on the diffuse-weighted image in the territory of the right middle cerebral artery. (B) FLAIR sequence with slight representation of the compromised territory without hemorrhagic transformation or contrast uptake. FLAIR, fluid-attenuated inversion recovery

Acute lymphoblastic leukemia (ALL) type chemotherapy was started with the HyperCVAD protocol and biweekly intrathecal chemotherapies. During his evaluation, the patient presented post-chemotherapy aplasia and multiple infectious complications due to invasive aspergillosis, pyogenic liver abscesses, and perforated appendicitis. On day 23 of induction chemotherapy, he presented with paresthesia in the tongue and pharynx accompanied by difficulty swallowing. Magnetic resonance angiography was performed, documenting a right frontoparietal subcortical lesion with poorly defined, irregular borders, not clearly vascular (Figure 3), suspicious for infiltration. Carotid Doppler and transesophageal echocardiogram found no pathological findings. The neurological deficit correlated with an improvement in magnetic resonance imaging (MRI) resolved after six intrathecal methotrexate chemotherapies, and FCM in CSF for BPDCN was negative. At the end of the HyperCVAD induction protocol, the response in bone marrow showed a measurable residual disease in FCM of 0.06% of blasts CD123 (+), CD56 (+), CD4 (+), CD34 (-), weak CD45 (+), CD7 (-), HLA-DR (+), CD38 (-), and CD22 (-).

Two weeks later, the neurological signs worsened, developing cauda equina syndrome (overflow urination requiring intermittent catheterization, sensory alterations in the perineal region and lower limbs, and lower limb weakness). CSF and nuclear MRI of the brain and spine with contrast were performed again, which revealed enhancement of the roots of the cauda equina, with negative CSF for infiltration by FCM. It was considered that the patient presented a progression of his disease in the peripheral nervous system and possibly hepatic infiltration, and therefore second-line treatment was started with an acute myeloid leukemia (AML) type chemotherapy protocol “7 + 3 (cytarabine with idarubicin)” associated with venetoclax from days 1 to 12, achieving a complete response with negative measurable residual bone marrow disease and improvement of neurological manifestations and possibly hepatic infiltration by imaging. The CSF remained negative for leukemic infiltration. The patient was considered a candidate for consolidation with HSCT. While transplantation was prepared, he received two cycles of additional consolidation with a cytarabine regimen with idarubicin (3+1) plus venetoclax on days 1 to 14. He is currently at his third month post-haploidentical bone marrow transplantation of a sibling. As a complication, he presented with grade 1 dermatological graft versus host disease (GVHD) and cytomegalovirus reactivation. He has stable blood cell count and remains on immunosuppressive therapy (methotrexate and tacrolimus) without clinical or laboratory data of relapse.

## Discussion

BPDCN is an uncommon hematological disease derived from plasmacytoid dendritic cells whose myeloid lineage was established in 2008. In 2016, the World Health Organization designated BPDCN to be in its own separate category within the myeloid class of neoplasms [5].

One of the largest series reported is the French group of Garnache-Ottou et al. with a total of 86 patients [6]. They found a mean age of diagnosis of 64 years with a male-to-female ratio of 4:1. Recent publications refer to a bimodal distribution with the first peak around 20 years and the second after 60 years, as has traditionally been described [7]. There seem to be two patterns of the disease: first and most prevalent one is the presence of single or multiple nodular skin lesions, plaques, or patches with subsequent dissemination of the disease in up to 90% of patients, while in the remaining 10%, a presentation of leukemic characteristics without skin involvement is observed, as was the case in our patient. Other visceral involvements include lymph nodes in up to 50%, CNS in 30% (a spectrum from no neurological signs to localized deficit), and liver/spleen in 20%, all of which were present in our case [3-8].

The morphology is not specific, but it is a first step that guides the diagnosis. Intermediate-sized blasts with peripheral nuclei and open chromatin and several nucleoli are usually found, without granules in the cytoplasm [9]. The diagnosis requires FMC or IHC and is challenging since there is no specific marker and they need to be assessed altogether. Initially, the presence of CD4 and CD56 should be evaluated since only 8% of BPDCNs are negative for these, and their mere presence without other specific lineage markers such as CD19, cCD3, MPO, CD14, or CD64 with high HLA-DR should alert about the possibility of this type of neoplasm [1]. The next step is to evaluate specific dendritic cell antigens such as CD123, TCL1, CD303, and CD304 [6]. Within the differential diagnosis, there is an entity known as proliferation of mature plasmacytoid dendritic cells associated with myeloid neoplasms (such as chronic myelomonocytic leukemia, myelodysplasia, and acute leukemias) characterized by irregular aggregates in bone marrow or lymph nodes of phenotypic plasmacytoid dendritic cells and morphologically similar to normal, with a Ki-67 proliferation index of less than 10% and almost always CD56 negative [3]. There is no genetic abnormality specific to the disease, but deletions of 5q21, 5q34, or 12p13 are described in more than half of the patients, none of which were found in our patient [6]. The reported gain (1p36) is not among the commonly described abnormalities, although this does not seem to have prognostic importance. The MYC translocation detected by FISH (fluorescence in situ hybridization) has a more aggressive behavior and is associated with a better response to chemotherapy protocols for ALL [1].

Historically, it has been a disease with a poor prognosis with a mean survival close to eight months in patients refractory to conventional chemotherapy and who do not receive consolidation with HSCT [10]. There is no consensus about the most appropriate chemotherapy, and in 2003, Reimer et al. presented data from 97 patients where no differences in response were detected according to the protocol used (lymphoma-type protocols such as CHOP vs. acute leukemia-type protocols), although maintenance of remission favored acute leukemia-type regimens with consolidation with HSCT with a median survival of 38.5 months [11]. In 2013, Pagano et al. published a series of 41 patients who received AML (63%) and ALL (37%) type chemotherapies, with the latter achieving a higher response rate [12]. The use of stricter CNS prophylaxis in these regimens has been proposed as an explanation for this better response.

The emergence of directed therapies targeting CD123 has revolutionized the natural history of the disease. CD123 is a transmembrane glycoprotein, also known as IL3 receptor, expressed in less than 1% of non-pathological cells [4]. Tagraxofusp is a truncated human IL3-diphtheria toxin fusion protein approved by the FDA in December 2018 after promising phase I studies that subsequently led to the largest multicenter study in patients with BPDCN conducted by Pemmaraju et al. in 47 people over four years, excluding patients with CNS involvement and those with albumin less than 3.2 g/dL due to the risk of capillary leak syndrome. The mean age was 68 years. The response rate was 90% and survival at 18 and 24 months was 59% and 52%, respectively, in patients who did not undergo transplantation. In 45% who received consolidation treatment with HSCT, median survival of 929 days was documented [13-14].

The use of HyperCVAD as a first-line regimen is supported by recent evidence also published by Pemmaraju et al., in 2021, with a retrospective series of 100 patients from 1999 to 2020, of which 35 received any combination of this regimen, 37 received tagraxofusp, and 28 received other treatments. Despite the methodological limitations, it calls to attention that the patients who received the HyperCVAD regimen achieved a higher complete remission (80% vs. 59% vs. 43%, p=0.01), although with no differences in overall survival (OS) and with similar post-HSCT outcomes [15]. In this way, it is concluded that HyperCVAD-type chemotherapy schemes can continue to be considered first-line treatment, and prospective studies are required to evaluate the comparative efficacy of other treatments such as tagraxofusp in monotherapy or in combination with traditional chemotherapy.

There is consensus that if a favorable outcome occurs to any of the schemes and the patient is suitable, it is preferred to consolidate with allogeneic HSCT, which has been the most extensively studied and has shown greater survival compared to autologous transplant [16]. In 2021, Bashir et al. provided new data on the consolidation of HSCT treatment in 17 patients who had a mean age of 39 years. Ten patients were in their first complete remission, and progression-free survival (PFS) at two and five years was 49% and 39%, respectively, and OS at two and five years was 65 and 40%, respectively. When evaluating PFS and OS in transplanted patients in the first remission, it was 80% vs 0% in patients who were transplanted at a later time, which highlights the importance of performing HSCT in first remission to achieve better results [17].

CNS involvement can reach up to 30% at the time of diagnosis and 100% in relapses, as described in a series of 13 patients by Martín-Martín et al., who did not initially present abnormalities on neurological physical examination but at the time of relapse had overt disease with seizures and cranial nerve involvement [18]. This has led to the appearance of recommendations on early lumbar puncture at diagnosis and consideration of schemes that include prophylactic intrathecal chemotherapy. In 2021, Greenwell et al. presented a cohort of 11 patients with CNS involvement managed with tagraxofusp; five patients received anti-CD123 therapy without achieving prevention of CNS involvement requiring intrathecal chemotherapy in all cases with BPDCN clearance in the FMC after the third dose. These findings suggest that intrathecal prophylaxis should be considered in all patients including those on targeted therapies [19].

Multiple clinical studies are underway with drugs directed at different therapeutic targets, in monotherapy or combination, including CART therapy directed at CD123, and treatment in patients with disease resistant to tagraxofusp with hypomethylating agents and venetoclax [20]. In our case, due to availability and reports of its efficacy in monotherapy or combination, the patient was offered management with venetoclax, an inhibitor of the antiapoptotic protein Bcl-2 widely expressed in BPDCN. There is currently a clinical trial underway (ClinicalTrials.gov: NCT03485547) that will allow us to establish safety, dosage, and possible combinations.

## Conclusions

Despite its rarity, it is necessary to recognize this neoplasm as quickly as possible due to its aggressive nature. As in most rare diseases, there is still uncertainty about what is the better treatment, but in recent years new drugs with significant activity against this disease have emerged that must be adequately compared with currently used schemes, and their combination with current protocols including transplantation should also be evaluated.
